# War, Alcohol, and Criminals

**Published:** 1917-01-20

**Authors:** 


### The Hospital

The Workers' Newspaper of Administrative Medicine and Institutional Life, Administration, National Insurance and Health.

No. 1598, Vol. LXI. SATURDAY, JANUARY 20, 1917. PRICE ONE PENNY

### WAR, ALCOHOL, AND CRIMINALS.

The Report of the Commissioners of Prisons and the Directors of Convict Prisons for the year ending March 31, 1916, contains some paragraphs which have a special social and national interest in view of their relation to the great question of the day and hour. Patriotism and prisons are not usually associated in our ordinary habits of thought, as the unwilling inhabitants of prisons are persons whose lives have shown the dominating influence of selfish and anti-social tendencies. But a simple and dogmatic classification of human beings, though convenient enough for the maker of labels, has never had a substantial basis of fact, and men and women, more especially under the influence of some powerful stimulus, show a complexity which defies the grasp of rigid definitions. The touch of nature occurs and the whole world is found to be akin. Thus the differences between those of us who are at liberty and our fellows who suffer restrictions in this respect are not so radical as the mere statement of the facts suggests, and "but for the grace of God" some of us perchance who are outside the prison walls might well have been within them. However this may be, we can hardly fail to read with some sense of generous emotion the announcement that "there is every reason to believe that the country's call for men appealed as strongly to the criminal as to other classes." Apart from many known instances of habitual criminals who have joined the Army and fought bravely for their country's cause, the conclusion just stated has the support of some very impressive figures. These show that the actual number of prisoners of military age received during the year covered by the present report was 19,169, as compared with the 61,739 registered in the 1914 report, and of these without question many would be physically unfit for military duty. Doubtless here, as elsewhere, men have acted under the influence of mixed motives, but all the same there remains the broad fact that to a lofty appeal there has been a worthy response even from many who have shown themselves enemies of society and exponents of the predatory instincts. The moral seems to be that some corresponding claim, in times of peace, could it only be found, might have an equal measure of success.

With such a disturbance of ordinary conditions as is indicated by the figures here quoted there is in many respects no sure ground of comparison between the records of the present report and those of former years. There are, in addition, other disturbing influences, and some of these are difficult to identify. Thus there has been a considerable decrease in the total of habitual drunkards committed to inebriate reformatories, and this in spite of the fact that the number of convictions for drunkenness registered against one and the same person has much increased. These last-mentioned figures have led to some confusion of thought, and have been quoted under the belief that they imply the extension of excessive drinking, more especially among women. So far as figures go, the very opposite of this is the case. Take, as an example, the records of Holloway Prison. These show that whereas in 1913 the women prisoners committed for drunkenness numbered 1,092, the figures of 1915 had fallen to 813. But the number of convictions during the same period had increased from 2,768 to 4,189—that is, in other words, the individual prisoners had diminished, but the average number of convictions per individual had increased. The last-mentioned record is one of 2.6 convictions per individual in 1913, increased to rather over 5.0 in 1916.

As we have already indicated, the figures to which reference is here made have been the subject of a misapprehension, and an increase in the number of convictions has been treated as an increase in the number of persons convicted. Still further, this alleged development has been utilised by those who claim that the only safety for the nation in the present juncture of affairs is complete suppression of the trade in alcoholic liquors. Some of the persons who urge this prohibition believe in their policy as a matter of principle and would have applied it in peace-time had they had the chance so to do. But others advocate it as justified by the present stress and suitable to the special conditions created by the war. For our own part we cannot accept such a conclusion. Of course we are heartily in favour of any conditions which will restrict or suppress excessive drinking, just as we are wholehearted supporters of the nation's fight for freedom from the tyranny of a military oligarchy. But we are satisfied that, many of those who are at present engaged in hard manual labour in the nation's interest, are not the worse, but the better, for the moderate supplies of malt liquor which they consume. If in particular districts there is spirit-drinking in such a degree as to hinder work of national importance, we have nothing to say against a claim for prohibition. But to attempt to alter, by an arbitrary decree, such a widespread habit as the drinking of beer is, we believe, neither necessary nor wise. The example of the French is sometimes quoted to us, but those who use this argument do not always explain that in France "alcohol" does not include wine, and that not a few civilians, as well as soldiers, place a high value on the wine ration which is supplied to the troops, whose courage and endurance will live for ever in the annals of their country. Restrictions which hinder abuse have our full sympathy, but good malt liquor in moderate quantities is helpful, if not necessary, to many workers, and this is a consideration which must not be ignored.

